# Supplementary material for: Iterative improvement in the automatic modular design of robot swarms
Source: PeerJ Comput Sci. 2020 Dec 7;6:e322. doi: 10.7717/peerj-cs.322 (PMC7924708; doi:10.7717/peerj-cs.322)
Supplement: Supplemental Information 3 [file peerj-cs-06-322-s003.zip › argos3/doc/api/standalone/a00654_source.html]

ARGoS: plugins/simulator/visualizations/qt-opengl/qtopengl\_main\_window.cpp Source File


- Main Page
- Related Pages
- Namespaces
- Classes
- Files

- File List
- File Members

# plugins/simulator/visualizations/qt-opengl/qtopengl\_main\_window.cpp

Go to the documentation of this file.

```
00001 
00007 #include "qtopengl_widget.h"
00008 #include "qtopengl_log_stream.h"
00009 #include "qtopengl_user_functions.h"
00010 #include "qtopengl_main_window.h"
00011 
00012 #include <argos3/core/config.h>
00013 #include <argos3/core/utility/plugins/dynamic_loading.h>
00014 #include <argos3/core/utility/logging/argos_log.h>
00015 #include <argos3/core/simulator/simulator.h>
00016 #include <argos3/core/simulator/loop_functions.h>
00017 
00018 #include <QtCore/QVariant>
00019 #include <QtWidgets/QAction>
00020 #include <QtWidgets/QApplication>
00021 #include <QtWidgets/QDockWidget>
00022 #include <QtWidgets/QHeaderView>
00023 #include <QtWidgets/QLCDNumber>
00024 #include <QtWidgets/QPushButton>
00025 #include <QtWidgets/QSpinBox>
00026 #include <QtWidgets/QDoubleSpinBox>
00027 #include <QtWidgets/QStatusBar>
00028 #include <QtWidgets/QWidget>
00029 #include <QLabel>
00030 #include <QCloseEvent>
00031 #include <QMessageBox>
00032 #include <QDir>
00033 #include <QFile>
00034 #include <QTextStream>
00035 #include <QToolBar>
00036 #include <QLayout>
00037 #include <QMenuBar>
00038 #include <QSettings>
00039 
00040 namespace argos {
00041 
00042    /****************************************/
00043    /****************************************/
00044 
00045    class CQTOpenGLLayout : public QLayout {
00046 
00047    public:
00048 
00049       CQTOpenGLLayout() :
00050          m_pcQTOpenGLItem(NULL) {
00051          setContentsMargins(0, 0, 0, 0);
00052       }
00053 
00054       virtual ~CQTOpenGLLayout() {
00055          if(m_pcQTOpenGLItem != NULL) {
00056             delete m_pcQTOpenGLItem;
00057          }
00058       }
00059 
00060       virtual void addItem(QLayoutItem* item) {
00061          if(m_pcQTOpenGLItem != NULL) {
00062             delete m_pcQTOpenGLItem;
00063          }
00064          m_pcQTOpenGLItem = item;
00065       }
00066       virtual int count() const {
00067          return (m_pcQTOpenGLItem != NULL) ? 1 : 0;
00068       }
00069 
00070       virtual QLayoutItem* itemAt(int index) const {
00071          return (index == 0) ? m_pcQTOpenGLItem : NULL;
00072       }
00073 
00074       virtual QLayoutItem* takeAt(int index) {
00075          if(index == 0) {
00076             QLayoutItem* pcRetVal = m_pcQTOpenGLItem;
00077             m_pcQTOpenGLItem = NULL;
00078             return pcRetVal;
00079          }
00080          else {
00081             return NULL;
00082          }
00083       }
00084 
00085       virtual QSize minimumSize () const {
00086          return QSize(320,240);
00087       }
00088 
00089       virtual QSize sizeHint () const {
00090          return QSize(640,480);
00091       }
00092 
00093       virtual void setGeometry(const QRect& r) {
00094          /* Set the layout geometry */
00095          QLayout::setGeometry(r);
00096          if(m_pcQTOpenGLItem != NULL) {
00097             /* Calculate the candidate sizes for the QTOpenGL widget */
00098             /* One is height-driven */
00099             QRect cCandidate1(r.x(), r.y(), (r.height() * 4) / 3, r.height());
00100             /* The other is width-driven */
00101             QRect cCandidate2(r.x(), r.y(), r.width(), (r.width() * 3) / 4);
00102             /* Pick the one that fits the rectangle better */
00103             if(r.contains(cCandidate1)) {
00104                /* Horizontal padding needed */
00105                int nPadding = (r.width() - cCandidate1.width()) / 2;
00106                cCandidate1.translate(nPadding, 0);
00107                m_pcQTOpenGLItem->setGeometry(cCandidate1);
00108             }
00109             else {
00110                /* Vertical padding needed */
00111                int nPadding = (r.height() - cCandidate2.height()) / 2;
00112                cCandidate2.translate(0, nPadding);
00113                m_pcQTOpenGLItem->setGeometry(cCandidate2);
00114             }
00115          }
00116       }
00117 
00118    private:
00119 
00120       QLayoutItem* m_pcQTOpenGLItem;
00121 
00122    };
00123 
00124    /****************************************/
00125    /****************************************/
00126 
00127    CQTOpenGLMainWindow::CQTOpenGLMainWindow(TConfigurationNode& t_tree) :
00128       m_pcUserFunctions(NULL) {
00129       /* Main window settings */
00130       std::string strTitle;
00131       GetNodeAttributeOrDefault<std::string>(t_tree, "title", strTitle, "ARGoS v" ARGOS_VERSION "-" ARGOS_RELEASE);
00132       setWindowTitle(tr(strTitle.c_str()));
00133       /* Restore settings, if any */
00134       ReadSettingsPreCreation();
00135       /* Add a status bar */
00136       m_pcStatusbar = new QStatusBar(this);
00137       setStatusBar(m_pcStatusbar);
00138       /* Create actions */
00139       CreateExperimentActions();
00140       CreateCameraActions();
00141       // CreatePOVRayActions();
00142       CreateHelpActions();
00143       /* Create user functions */
00144       CreateUserFunctions(t_tree);
00145       /* Create the central widget */
00146       CreateOpenGLWidget(t_tree);
00147       /* Create menus */
00148       CreateExperimentMenu();
00149       CreateCameraMenu();
00150       // CreatePOVRayMenu();
00151       CreateHelpMenu();
00152       /* Create toolbars */
00153       CreateExperimentToolBar();
00154       CreateCameraToolBar();
00155       /* Create the message dock window */
00156       CreateLogMessageDock();
00157       /* Restore settings, if any */
00158       ReadSettingsPostCreation();
00159       /* Creates the signal/slot connections */
00160       CreateConnections();
00161       /* Set experiment state */
00162       m_eExperimentState = EXPERIMENT_INITIALIZED;
00163       /* Should we play instantly? */
00164       bool bAutoPlay = false;
00165       GetNodeAttributeOrDefault(t_tree, "autoplay", bAutoPlay, bAutoPlay);
00166       if(bAutoPlay) {
00167          PlayExperiment();
00168       }
00169    }
00170 
00171    /****************************************/
00172    /****************************************/
00173 
00174    CQTOpenGLMainWindow::~CQTOpenGLMainWindow() {
00175       delete m_pcUserFunctions;
00176       delete m_pcLogStream;
00177       delete m_pcLogErrStream;
00178       if(m_bWasLogColored) {
00179          LOG.EnableColoredOutput();
00180          LOGERR.EnableColoredOutput();
00181       }
00182    }
00183 
00184    /****************************************/
00185    /****************************************/
00186 
00187    void CQTOpenGLMainWindow::ReadSettingsPreCreation() {
00188       QSettings cSettings;
00189       cSettings.beginGroup("MainWindow");
00190       resize(cSettings.value("size", QSize(640,480)).toSize());
00191       move(cSettings.value("position", QPoint(0,0)).toPoint());
00192       if(cSettings.contains("icon_dir")) {
00193          m_strIconDir = cSettings.value("icon_dir").toString();
00194          if(m_strIconDir.at(m_strIconDir.length()-1) != '/') {
00195             m_strIconDir.append("/");
00196          }
00197       }
00198       else {
00199          m_strIconDir = QString::fromStdString(CSimulator::GetInstance().GetInstallationDirectory());
00200          m_strIconDir += "/include/argos3/plugins/simulator/visualizations/qt-opengl/icons/";
00201       }
00202       if(cSettings.contains("texture_dir")) {
00203          m_strTextureDir = cSettings.value("texture_dir").toString();
00204          if(m_strTextureDir.at(m_strTextureDir.length()-1) != '/') {
00205             m_strTextureDir.append("/");
00206          }
00207       }
00208       else {
00209          m_strTextureDir = QString::fromStdString(CSimulator::GetInstance().GetInstallationDirectory());
00210          m_strTextureDir += "/include/argos3/plugins/simulator/visualizations/qt-opengl/textures/";
00211       }
00212       cSettings.endGroup();
00213    }
00214 
00215    /****************************************/
00216    /****************************************/
00217 
00218    void CQTOpenGLMainWindow::ReadSettingsPostCreation() {
00219       QSettings cSettings;
00220       cSettings.beginGroup("MainWindow");
00221       restoreState(cSettings.value("docks").toByteArray());
00222       cSettings.endGroup();
00223    }
00224 
00225    /****************************************/
00226    /****************************************/
00227 
00228    void CQTOpenGLMainWindow::WriteSettings() {
00229       QSettings cSettings;
00230       cSettings.beginGroup("MainWindow");
00231       cSettings.setValue("docks", saveState());
00232       cSettings.setValue("size", size());
00233       cSettings.setValue("position", pos());
00234       cSettings.setValue("icon_dir", m_strIconDir);
00235       cSettings.setValue("texture_dir", m_strTextureDir);
00236       cSettings.endGroup();
00237    }
00238 
00239    /****************************************/
00240    /****************************************/
00241 
00242    void CQTOpenGLMainWindow::CreateExperimentActions() {
00243       /* Add the play action */
00244       QIcon cPlayIcon;
00245       cPlayIcon. addPixmap(QPixmap(m_strIconDir + "/play.png"));
00246       m_pcPlayAction = new QAction(cPlayIcon, tr("&Play"), this);
00247       m_pcPlayAction->setShortcut(Qt::Key_P);
00248       m_pcPlayAction->setToolTip(tr("Play experiment"));
00249       m_pcPlayAction->setStatusTip(tr("Play experiment"));
00250       if(! CSimulator::GetInstance().IsRealTimeClock()) {
00251          /* Add the step action */
00252          QIcon cStepIcon;
00253          cStepIcon.addPixmap(QPixmap(m_strIconDir + "/step.png"));
00254          m_pcStepAction = new QAction(cStepIcon, tr("&Step"), this);
00255          m_pcStepAction->setToolTip(tr("Step experiment"));
00256          m_pcStepAction->setStatusTip(tr("Step experiment"));
00257          m_pcStepAction->setShortcut(Qt::Key_X);
00258          /* Add the fast forward action */
00259          QIcon cFastForwardIcon;
00260          cFastForwardIcon.addPixmap(QPixmap(m_strIconDir + "/fast_forward.png"));
00261          m_pcFastForwardAction = new QAction(cFastForwardIcon, tr("&Fast Forward"), this);
00262          m_pcFastForwardAction->setToolTip(tr("Fast forward experiment"));
00263          m_pcFastForwardAction->setStatusTip(tr("Fast forward experiment"));
00264          m_pcFastForwardAction->setShortcut(Qt::Key_F);
00265          /* Add the pause action */
00266          QIcon cPauseIcon;
00267          cPauseIcon. addPixmap(QPixmap(m_strIconDir + "/pause.png"));
00268          m_pcPauseAction = new QAction(cPauseIcon, tr("&Pause"), this);
00269          m_pcPauseAction->setShortcut(Qt::Key_O);
00270          m_pcPauseAction->setToolTip(tr("Pause experiment"));
00271          m_pcPauseAction->setStatusTip(tr("Pause experiment"));
00272          m_pcPauseAction->setEnabled(false);
00273       }
00274       /* Add the terminate action */
00275       QIcon cTerminateIcon;
00276       cTerminateIcon.addPixmap(QPixmap(m_strIconDir + "/stop.png"));
00277       m_pcTerminateAction = new QAction(cTerminateIcon, tr("&Terminate"), this);
00278       m_pcTerminateAction->setShortcut(Qt::Key_T);
00279       m_pcTerminateAction->setToolTip(tr("Terminate experiment"));
00280       m_pcTerminateAction->setStatusTip(tr("Terminate experiment"));
00281       m_pcTerminateAction->setEnabled(false);
00282       /* Add the reset action */
00283       QIcon cResetIcon;
00284       cResetIcon.addPixmap(QPixmap(m_strIconDir + "/reset.png"));
00285       m_pcResetAction = new QAction(cResetIcon, tr("&Reset"), this);
00286       m_pcResetAction->setToolTip(tr("Reset experiment"));
00287       m_pcResetAction->setStatusTip(tr("Reset experiment"));
00288       m_pcResetAction->setShortcut(Qt::Key_R);
00289       m_pcResetAction->setEnabled(false);
00290       /* Add the capture action */
00291       QIcon cCaptureIcon;
00292       cCaptureIcon.addPixmap(QPixmap(m_strIconDir + "/record.png"));
00293       m_pcCaptureAction = new QAction(cCaptureIcon, tr("&Capture"), this);
00294       m_pcCaptureAction->setToolTip(tr("Capture frames"));
00295       m_pcCaptureAction->setStatusTip(tr("Capture frames"));
00296       m_pcCaptureAction->setCheckable(true);
00297       m_pcCaptureAction->setShortcut(Qt::Key_C);
00298       /* Add the quit action */
00299       m_pcQuitAction = new QAction(tr("&Quit"), this);
00300       m_pcQuitAction->setStatusTip(tr("Quit the simulator"));
00301    }
00302 
00303    /****************************************/
00304    /****************************************/
00305 
00306    void CQTOpenGLMainWindow::CreateCameraActions() {
00307       /* Add the switch camera buttons */
00308       m_pcSwitchCameraActionGroup = new QActionGroup(this);
00309       QIcon cCameraIcon;
00310       cCameraIcon.addPixmap(QPixmap(m_strIconDir + "/camera.png"));
00311       for(UInt32 i = 0; i < 12; ++i) {
00312          QAction* pcAction = new QAction(cCameraIcon, tr(QString("Camera %1").arg(i+1).toLatin1().data()), m_pcSwitchCameraActionGroup);
00313          pcAction->setToolTip(tr(QString("Switch to camera %1").arg(i+1).toLatin1().data()));
00314          pcAction->setStatusTip(tr(QString("Switch to camera %1").arg(i+1).toLatin1().data()));
00315          pcAction->setCheckable(true);
00316          pcAction->setShortcut(Qt::Key_F1 + i);
00317          pcAction->setData(i);
00318          m_pcSwitchCameraActions.push_back(pcAction);
00319       }
00320       m_pcSwitchCameraActions.first()->setChecked(true);
00321       /* Add the show camera XML button */
00322       m_pcShowCameraXMLAction = new QAction(tr("&Show XML..."), this);
00323       m_pcShowCameraXMLAction->setStatusTip(tr("Show XML configuration for all cameras"));
00324    }
00325 
00326    /****************************************/
00327    /****************************************/
00328 
00329    // void CQTOpenGLMainWindow::CreatePOVRayActions() {
00330    //    /* Add the POVRay XML button */
00331    //    QIcon cPOVRayXMLIcon;
00332    //    m_pcPOVRayXMLAction = new QAction(cPOVRayXMLIcon, tr("&Edit XML"), this);
00333    //    m_pcPOVRayXMLAction->setToolTip(tr("Edit POV-Ray XML configuration"));
00334    //    m_pcPOVRayXMLAction->setStatusTip(tr("Edit POV-Ray XML configuration"));
00335    //    /* Add the POVRay Preview button */
00336    //    QIcon cPOVRayPreviewIcon;
00337    //    m_pcPOVRayPreviewAction = new QAction(cPOVRayPreviewIcon, tr("&Preview"), this);
00338    //    m_pcPOVRayPreviewAction->setToolTip(tr("Preview POV-Ray rendering of this scene"));
00339    //    m_pcPOVRayPreviewAction->setStatusTip(tr("Preview POV-Ray rendering of this scene"));
00340    // }
00341 
00342    /****************************************/
00343    /****************************************/
00344 
00345    void CQTOpenGLMainWindow::CreateHelpActions() {
00346       /* Add the 'about qt' button */
00347       m_pcAboutQTAction = new QAction(tr("About &Qt"), this);
00348       m_pcAboutQTAction->setStatusTip(tr("Show the Qt library's About box"));
00349    }
00350 
00351    /****************************************/
00352    /****************************************/
00353 
00354    void CQTOpenGLMainWindow::CreateExperimentToolBar() {
00355       m_pcExperimentToolBar = addToolBar(tr("Experiment"));
00356       m_pcExperimentToolBar->setObjectName("ExperimentToolBar");
00357       m_pcExperimentToolBar->setIconSize(QSize(32,32));
00358       m_pcCurrentStepLCD = new QLCDNumber(m_pcExperimentToolBar);
00359       m_pcCurrentStepLCD->setToolTip(tr("Current step"));
00360       m_pcCurrentStepLCD->setDigitCount(6);
00361       m_pcCurrentStepLCD->setSegmentStyle(QLCDNumber::Flat);
00362       m_pcExperimentToolBar->addWidget(m_pcCurrentStepLCD);
00363       m_pcExperimentToolBar->addSeparator();
00364       if(! CSimulator::GetInstance().IsRealTimeClock()) {
00365          m_pcExperimentToolBar->addAction(m_pcStepAction);
00366       }
00367       m_pcExperimentToolBar->addAction(m_pcPlayAction);
00368       if(! CSimulator::GetInstance().IsRealTimeClock()) {
00369          m_pcExperimentToolBar->addAction(m_pcPauseAction);
00370          m_pcExperimentToolBar->addAction(m_pcFastForwardAction);
00371          m_pcDrawFrameEvery = new QSpinBox(m_pcExperimentToolBar);
00372          m_pcDrawFrameEvery->setToolTip(tr("Draw frame every X steps when in fast-forward"));
00373          m_pcDrawFrameEvery->setMinimum(1);
00374          m_pcDrawFrameEvery->setMaximum(999);
00375          m_pcDrawFrameEvery->setValue(1);
00376          m_pcExperimentToolBar->addWidget(m_pcDrawFrameEvery);
00377       }
00378       m_pcExperimentToolBar->addSeparator();
00379       m_pcExperimentToolBar->addAction(m_pcTerminateAction);
00380       m_pcExperimentToolBar->addAction(m_pcResetAction);
00381       m_pcExperimentToolBar->addSeparator();
00382       m_pcExperimentToolBar->addAction(m_pcCaptureAction);
00383    }
00384 
00385    /****************************************/
00386    /****************************************/
00387 
00388    void CQTOpenGLMainWindow::CreateExperimentMenu() {
00389       m_pcExperimentMenu = menuBar()->addMenu(tr("&Experiment"));
00390       m_pcExperimentMenu->addAction(m_pcPlayAction);
00391       if(! CSimulator::GetInstance().IsRealTimeClock()) {
00392          m_pcExperimentMenu->addAction(m_pcPauseAction);
00393          m_pcExperimentMenu->addAction(m_pcFastForwardAction);
00394          m_pcExperimentMenu->addAction(m_pcStepAction);
00395       }
00396       m_pcExperimentMenu->addSeparator();
00397       m_pcExperimentMenu->addAction(m_pcTerminateAction);
00398       m_pcExperimentMenu->addAction(m_pcResetAction);
00399       m_pcExperimentMenu->addSeparator();
00400       m_pcExperimentMenu->addAction(m_pcCaptureAction);
00401       m_pcExperimentMenu->addSeparator();
00402       m_pcExperimentMenu->addAction(m_pcQuitAction);
00403    }
00404 
00405    /****************************************/
00406    /****************************************/
00407 
00408    void CQTOpenGLMainWindow::CreateCameraToolBar() {
00409       m_pcCameraToolBar = new QToolBar(tr("Camera"));
00410       m_pcCameraToolBar->setAllowedAreas(Qt::LeftToolBarArea |
00411                                          Qt::RightToolBarArea |
00412                                          Qt::BottomToolBarArea);
00413       m_pcCameraToolBar->setObjectName("CameraToolBar");
00414       m_pcCameraToolBar->setIconSize(QSize(32,32));
00415       m_pcCameraToolBar->addActions(m_pcSwitchCameraActions);
00416       m_pcCameraToolBar->addSeparator();
00417       m_pcFocalLength = new QDoubleSpinBox(m_pcCameraToolBar);
00418       m_pcFocalLength->setToolTip(tr("Set the focal length of the current camera"));
00419       m_pcFocalLength->setSuffix("mm");
00420       m_pcFocalLength->setDecimals(1);
00421       m_pcFocalLength->setSingleStep(1.0f);
00422       m_pcFocalLength->setRange(1.0f, 999.0f);
00423       m_pcFocalLength->setValue(m_pcOpenGLWidget->GetCamera().GetSetting(0).LensFocalLength * 1000.0f);
00424       m_pcCameraToolBar->addWidget(m_pcFocalLength);
00425       addToolBar(Qt::LeftToolBarArea, m_pcCameraToolBar);
00426    }
00427 
00428    /****************************************/
00429    /****************************************/
00430 
00431    void CQTOpenGLMainWindow::CreateCameraMenu() {
00432       m_pcCameraMenu = menuBar()->addMenu(tr("&Camera"));
00433       m_pcCameraMenu->addActions(m_pcSwitchCameraActions);
00434       m_pcCameraMenu->addAction(m_pcShowCameraXMLAction);
00435    }
00436 
00437    /****************************************/
00438    /****************************************/
00439 
00440    // void CQTOpenGLMainWindow::CreatePOVRayMenu() {
00441    //    m_pcPOVRayMenu = menuBar()->addMenu(tr("&POVRay"));
00442    //    m_pcPOVRayMenu->addAction(m_pcPOVRayPreviewAction);
00443    //    m_pcPOVRayMenu->addAction(m_pcPOVRayXMLAction);
00444    // }
00445 
00446    /****************************************/
00447    /****************************************/
00448 
00449    void CQTOpenGLMainWindow::CreateHelpMenu() {
00450       m_pcHelpMenu = menuBar()->addMenu(tr("&?"));
00451       m_pcHelpMenu->addAction(m_pcAboutQTAction);
00452    }
00453 
00454    /****************************************/
00455    /****************************************/
00456 
00457    void CQTOpenGLMainWindow::CreateOpenGLWidget(TConfigurationNode& t_tree) {
00458       /* Create the surface format */
00459       QSurfaceFormat cFormat = QSurfaceFormat::defaultFormat();
00460       cFormat.setSamples(4);
00461       cFormat.setDepthBufferSize(24);
00462       /* Create the widget */
00463       QWidget* pcPlaceHolder = new QWidget(this);
00464       m_pcOpenGLWidget = new CQTOpenGLWidget(pcPlaceHolder, *this, *m_pcUserFunctions);
00465       m_pcOpenGLWidget->setFormat(cFormat);
00466       m_pcOpenGLWidget->setCursor(QCursor(Qt::OpenHandCursor));
00467       m_pcOpenGLWidget->GetCamera().Init(t_tree);
00468       m_pcOpenGLWidget->GetFrameGrabData().Init(t_tree);
00469       /* Invert mouse controls? */
00470       bool bInvertMouse;
00471       GetNodeAttributeOrDefault(t_tree, "invert_mouse", bInvertMouse, false);
00472       m_pcOpenGLWidget->SetInvertMouse(bInvertMouse);
00473       /* Set the window as the central widget */
00474       CQTOpenGLLayout* pcQTOpenGLLayout = new CQTOpenGLLayout();
00475       pcQTOpenGLLayout->addWidget(m_pcOpenGLWidget);
00476       pcPlaceHolder->setLayout(pcQTOpenGLLayout);
00477       setCentralWidget(pcPlaceHolder);
00478       /* Initialize the user functions */
00479       if(NodeExists(t_tree, "user_functions")) {
00480          /* Use the passed user functions */
00481          /* Get data from XML */
00482          TConfigurationNode tNode = GetNode(t_tree, "user_functions");
00483          m_pcUserFunctions->Init(tNode);
00484       }
00485    }
00486 
00487    /****************************************/
00488    /****************************************/
00489 
00490    void CQTOpenGLMainWindow::CreateLogMessageDock() {
00491       /* Store the log color flag to be able to restore at exit */
00492       m_bWasLogColored = LOG.IsColoredOutput();
00493       /* Create a dockable window */
00494       m_pcLogDock = new QDockWidget(tr("Log"), this);
00495       m_pcLogDock->setObjectName("LogDockWindow");
00496       m_pcLogDock->setFeatures(QDockWidget::DockWidgetMovable |
00497                                QDockWidget::DockWidgetFloatable);
00498       m_pcLogDock->setAllowedAreas(Qt::LeftDockWidgetArea |
00499                                    Qt::RightDockWidgetArea |
00500                                    Qt::BottomDockWidgetArea);
00501       /* Create a textual window to be used as a buffer */
00502       m_pcDockLogBuffer = new QTextEdit();
00503       m_pcDockLogBuffer->setReadOnly(true);
00504       LOG.Flush(); /* Write all the pending stuff */
00505       LOG.DisableColoredOutput(); /* Colors are not necessary */
00506       m_pcDockLogBuffer->append("<b>[t=0]</b> Log started."); /* Write something in the buffer */
00507       /* Redirect stdout to the buffer */
00508       m_pcLogStream = new CQTOpenGLLogStream(LOG.GetStream(), m_pcDockLogBuffer);
00509       /* Add the dockable window to the main widget */
00510       m_pcLogDock->setWidget(m_pcDockLogBuffer);
00511       addDockWidget(Qt::RightDockWidgetArea, m_pcLogDock);
00512       /* Create a dockable window */
00513       m_pcLogErrDock = new QDockWidget(tr("LogErr"), this);
00514       m_pcLogErrDock->setObjectName("LogErrDockWindow");
00515       m_pcLogErrDock->setFeatures(QDockWidget::DockWidgetMovable |
00516                                   QDockWidget::DockWidgetFloatable);
00517       m_pcLogErrDock->setAllowedAreas(Qt::LeftDockWidgetArea |
00518                                       Qt::RightDockWidgetArea |
00519                                       Qt::BottomDockWidgetArea);
00520       /* Create a textual window to be used as a buffer */
00521       m_pcDockLogErrBuffer = new QTextEdit();
00522       m_pcDockLogErrBuffer->setReadOnly(true);
00523       LOGERR.Flush(); /* Write all the pending stuff */
00524       LOGERR.DisableColoredOutput(); /* Colors are not necessary */
00525       m_pcDockLogErrBuffer->append("<b>[t=0]</b> LogErr started."); /* Write something in the buffer */
00526       /* Redirect stderr to the buffer */
00527       m_pcLogErrStream = new CQTOpenGLLogStream(LOGERR.GetStream(), m_pcDockLogErrBuffer);
00528       m_pcLogErrDock->setWidget(m_pcDockLogErrBuffer);
00529       /* Add the dockable window to the main widget */
00530       addDockWidget(Qt::RightDockWidgetArea, m_pcLogErrDock);
00531 
00532    }
00533 
00534    /****************************************/
00535    /****************************************/
00536 
00537    void CQTOpenGLMainWindow::CreateConnections() {
00538       /* Play button pressed */
00539       connect(m_pcPlayAction, SIGNAL(triggered()),
00540               this, SLOT(PlayExperiment()));
00541       /* Reset button pressed */
00542       connect(m_pcResetAction, SIGNAL(triggered()),
00543               this, SLOT(ResetExperiment()));
00544       /* A experiment step has been completed */
00545       connect(m_pcOpenGLWidget, SIGNAL(StepDone(int)),
00546               m_pcCurrentStepLCD, SLOT(display(int)));
00547       /* The experiment has been completed */
00548       connect(m_pcOpenGLWidget, SIGNAL(ExperimentDone()),
00549               this, SLOT(TerminateExperiment()));
00550       /* The experiment has been completed */
00551       connect(m_pcTerminateAction, SIGNAL(triggered()),
00552               this, SLOT(TerminateExperiment()));
00553       if(! CSimulator::GetInstance().IsRealTimeClock()) {
00554          /* Pause button pressed */
00555          connect(m_pcPauseAction, SIGNAL(triggered()),
00556                  this, SLOT(PauseExperiment()));
00557          /* Step button pressed */
00558          connect(m_pcStepAction, SIGNAL(triggered()),
00559                  this, SLOT(StepExperiment()));
00560          /* Fast forward button pressed */
00561          connect(m_pcFastForwardAction, SIGNAL(triggered()),
00562                  this, SLOT(FastForwardExperiment()));
00563          /* 'Draw frame every' spin box value changed */
00564          connect(m_pcDrawFrameEvery, SIGNAL(valueChanged(int)),
00565                  m_pcOpenGLWidget, SLOT(SetDrawFrameEvery(int)));
00566       }
00567       // /* POV-Ray XML button pressed */
00568       // connect(m_pcPOVRayXMLAction, SIGNAL(triggered()),
00569       //         this, SLOT(POVRaySceneXMLPopUp()));
00570       // /* POV-Ray XML button pressed */
00571       // connect(m_pcPOVRayPreviewAction, SIGNAL(triggered()),
00572       //         this, SLOT(POVRayScenePreview()));
00573       /* Capture button toggled */
00574       connect(m_pcCaptureAction, SIGNAL(triggered(bool)),
00575               m_pcOpenGLWidget, SLOT(SetGrabFrame(bool)));
00576       /* Quit the simulator */
00577       connect(m_pcQuitAction, SIGNAL(triggered()),
00578               qApp, SLOT(quit()));
00579       /* Show the 'About Qt' dialog */
00580       connect(m_pcAboutQTAction, SIGNAL(triggered()),
00581               qApp, SLOT(aboutQt()));
00582       /* Toggle the camera */
00583       connect(m_pcSwitchCameraActionGroup, SIGNAL(triggered(QAction*)),
00584               this, SLOT(SwitchCamera(QAction*)));
00585       connect(this, SIGNAL(CameraSwitched(int)),
00586               m_pcOpenGLWidget, SLOT(SetCamera(int)));
00587       /* Camera focal length */
00588       connect(m_pcFocalLength, SIGNAL(valueChanged(double)),
00589               m_pcOpenGLWidget, SLOT(SetCameraFocalLength(double)));
00590       /* POV-Ray XML button pressed */
00591       connect(m_pcShowCameraXMLAction, SIGNAL(triggered()),
00592               this, SLOT(CameraXMLPopUp()));
00593    }
00594 
00595    /****************************************/
00596    /****************************************/
00597 
00598    void CQTOpenGLMainWindow::CreateUserFunctions(TConfigurationNode& t_tree) {
00599       /* Parse XML for user functions */
00600       if(NodeExists(t_tree, "user_functions")) {
00601          /* Use the passed user functions */
00602          /* Get data from XML */
00603          TConfigurationNode tNode = GetNode(t_tree, "user_functions");
00604          std::string strLabel, strLibrary;
00605          GetNodeAttribute(tNode, "label", strLabel);
00606          GetNodeAttributeOrDefault(tNode, "library", strLibrary, strLibrary);
00607          try {
00608             /* Load the library */
00609             if(strLibrary != "") {
00610                CDynamicLoading::LoadLibrary(strLibrary);
00611             }
00612             /* Create the user functions */
00613             m_pcUserFunctions = CFactory<CQTOpenGLUserFunctions>::New(strLabel);
00614             m_pcUserFunctions->SetMainWindow(*this);
00615          }
00616          catch(CARGoSException& ex) {
00617             THROW_ARGOSEXCEPTION_NESTED("Failed opening QTOpenGL user function library", ex);
00618          }
00619       }
00620       else {
00621          /* Use standard (empty) user functions */
00622          m_pcUserFunctions = new CQTOpenGLUserFunctions;
00623          m_pcUserFunctions->SetMainWindow(*this);
00624       }
00625    }
00626 
00627    /****************************************/
00628    /****************************************/
00629 
00630    void CQTOpenGLMainWindow::closeEvent(QCloseEvent* pc_event) {
00631       m_pcUserFunctions->Destroy();
00632       WriteSettings();
00633       pc_event->accept();
00634    }
00635 
00636    /****************************************/
00637    /****************************************/
00638 
00639    void CQTOpenGLMainWindow::PlayExperiment() {
00640       /* Make sure we are in the right state */
00641       if(m_eExperimentState != EXPERIMENT_INITIALIZED &&
00642          m_eExperimentState != EXPERIMENT_PAUSED) {
00643          LOGERR << "[BUG] CQTOpenGLMainWindow::PlayExperiment() called in wrong state: "
00644                 << m_eExperimentState
00645                 << std::endl;
00646          LOGERR.Flush();
00647          return;
00648       }
00649       /* Toggle action states */
00650       m_pcPlayAction->setEnabled(false);
00651       m_pcResetAction->setEnabled(false);
00652       m_pcTerminateAction->setEnabled(true);
00653       if(! CSimulator::GetInstance().IsRealTimeClock()) {
00654          m_pcPauseAction->setEnabled(true);
00655          m_pcFastForwardAction->setEnabled(false);
00656          m_pcStepAction->setEnabled(false);
00657       }
00658       /* Call OpenGL widget */
00659       m_pcOpenGLWidget->PlayExperiment();
00660       /* Change state and emit signals */
00661       m_eExperimentState = EXPERIMENT_PLAYING;
00662       if(m_eExperimentState == EXPERIMENT_INITIALIZED) {
00663          /* The experiment has just been started */
00664          emit ExperimentStarted();
00665       }
00666       emit ExperimentPlaying();
00667    }
00668 
00669    /****************************************/
00670    /****************************************/
00671 
00672    void CQTOpenGLMainWindow::FastForwardExperiment() {
00673       /* Make sure we are in the right state */
00674       if(m_eExperimentState != EXPERIMENT_INITIALIZED &&
00675          m_eExperimentState != EXPERIMENT_PAUSED) {
00676          LOGERR << "[BUG] CQTOpenGLMainWindow::FastForwardExperiment() called in wrong state: "
00677                 << m_eExperimentState
00678                 << std::endl;
00679          LOGERR.Flush();
00680          return;
00681       }
00682       /* Toggle action states */
00683       m_pcPlayAction->setEnabled(false);
00684       m_pcPauseAction->setEnabled(true);
00685       m_pcResetAction->setEnabled(false);
00686       m_pcTerminateAction->setEnabled(true);
00687       m_pcFastForwardAction->setEnabled(false);
00688       m_pcStepAction->setEnabled(false);
00689       /* Call OpenGL widget */
00690       m_pcOpenGLWidget->FastForwardExperiment();
00691       /* Change state and emit signals */
00692       m_eExperimentState = EXPERIMENT_FAST_FORWARDING;
00693       if(m_eExperimentState == EXPERIMENT_INITIALIZED) {
00694          /* The experiment has just been started */
00695          emit ExperimentStarted();
00696       }
00697       emit ExperimentFastForwarding();
00698    }
00699 
00700    /****************************************/
00701    /****************************************/
00702 
00703    void CQTOpenGLMainWindow::StepExperiment() {
00704       /* Make sure we are in the right state */
00705       if(m_eExperimentState != EXPERIMENT_INITIALIZED &&
00706          m_eExperimentState != EXPERIMENT_PAUSED) {
00707          LOGERR << "[BUG] CQTOpenGLMainWindow::StepExperiment() called in wrong state: "
00708                 << m_eExperimentState
00709                 << std::endl;
00710          LOGERR.Flush();
00711          return;
00712       }
00713       /* Toggle action states */
00714       m_pcPlayAction->setEnabled(true);
00715       m_pcResetAction->setEnabled(false);
00716       m_pcTerminateAction->setEnabled(true);
00717       m_pcFastForwardAction->setEnabled(true);
00718       m_pcStepAction->setEnabled(true);
00719       /* Call OpenGL widget */
00720       m_pcOpenGLWidget->StepExperiment();
00721       /* Change state and emit signals */
00722       m_eExperimentState = EXPERIMENT_PAUSED;
00723       emit ExperimentPaused();
00724    }
00725 
00726    /****************************************/
00727    /****************************************/
00728 
00729    void CQTOpenGLMainWindow::PauseExperiment() {
00730       /* Make sure we are in the right state */
00731       if(m_eExperimentState != EXPERIMENT_PLAYING &&
00732          m_eExperimentState != EXPERIMENT_FAST_FORWARDING) {
00733          LOGERR << "[BUG] CQTOpenGLMainWindow::PauseExperiment() called in wrong state: "
00734                 << m_eExperimentState
00735                 << std::endl;
00736          LOGERR.Flush();
00737          return;
00738       }
00739       /* Toggle action states */
00740       m_pcPlayAction->setEnabled(true);
00741       m_pcResetAction->setEnabled(false);
00742       m_pcTerminateAction->setEnabled(true);
00743       if(! CSimulator::GetInstance().IsRealTimeClock()) {
00744          m_pcPauseAction->setEnabled(false);
00745          m_pcFastForwardAction->setEnabled(true);
00746          m_pcStepAction->setEnabled(true);
00747       }
00748       /* Call OpenGL widget */
00749       m_pcOpenGLWidget->PauseExperiment();
00750       /* Change state and emit signals */
00751       m_eExperimentState = EXPERIMENT_PAUSED;
00752       emit ExperimentPaused();
00753    }
00754 
00755    /****************************************/
00756    /****************************************/
00757 
00758    void CQTOpenGLMainWindow::TerminateExperiment() {
00759       /* Make sure we are in the right state */
00760       if(m_eExperimentState != EXPERIMENT_PLAYING &&
00761          m_eExperimentState != EXPERIMENT_PAUSED &&
00762          m_eExperimentState != EXPERIMENT_FAST_FORWARDING &&
00763          m_eExperimentState != EXPERIMENT_SUSPENDED) {
00764          LOGERR << "[BUG] CQTOpenGLMainWindow::TerminateExperiment() called in wrong state: "
00765                 << m_eExperimentState
00766                 << std::endl;
00767          LOGERR.Flush();
00768          return;
00769       }
00770       /* Call OpenGL widget */
00771       m_pcOpenGLWidget->PauseExperiment();
00772       /* Toggle action states */
00773       m_pcPlayAction->setEnabled(false);
00774       m_pcResetAction->setEnabled(true);
00775       m_pcTerminateAction->setEnabled(false);
00776       m_pcCaptureAction->setEnabled(false);
00777       m_pcCaptureAction->setChecked(false);
00778       if(! CSimulator::GetInstance().IsRealTimeClock()) {
00779          m_pcPauseAction->setEnabled(false);
00780          m_pcStepAction->setEnabled(false);
00781          m_pcFastForwardAction->setEnabled(false);
00782       }
00783       /* Call ARGoS to terminate the experiment */
00784       CSimulator::GetInstance().Terminate();
00785       CSimulator::GetInstance().GetLoopFunctions().PostExperiment();
00786       LOG.Flush();
00787       LOGERR.Flush();
00788       /* Change state and emit signal */
00789       m_eExperimentState = EXPERIMENT_DONE;
00790       emit ExperimentDone();
00791    }
00792 
00793    /****************************************/
00794    /****************************************/
00795 
00796    void CQTOpenGLMainWindow::ResetExperiment() {
00797       /* Make sure we are in the right state */
00798       if(m_eExperimentState != EXPERIMENT_SUSPENDED &&
00799          m_eExperimentState != EXPERIMENT_DONE) {
00800          LOGERR << "[BUG] CQTOpenGLMainWindow::ResetExperiment() called in wrong state: "
00801                 << m_eExperimentState
00802                 << std::endl;
00803          LOGERR.Flush();
00804          return;
00805       }
00806       /* Toggle action states */
00807       m_pcPlayAction->setEnabled(true);
00808       m_pcResetAction->setEnabled(false);
00809       m_pcTerminateAction->setEnabled(false);
00810       m_pcCaptureAction->setEnabled(true);
00811       m_pcCaptureAction->setChecked(false);
00812       if(! CSimulator::GetInstance().IsRealTimeClock()) {
00813          m_pcPauseAction->setEnabled(false);
00814          m_pcStepAction->setEnabled(true);
00815          m_pcFastForwardAction->setEnabled(true);
00816       }
00817       /* Reset step counter and log */
00818       m_pcCurrentStepLCD->display(0);
00819       m_pcDockLogBuffer->setHtml("<b>[t=0]</b> Log restarted.");
00820       m_pcDockLogErrBuffer->setHtml("<b>[t=0]</b> LogErr restarted.");
00821       /* Call OpenGL widget */
00822       m_pcOpenGLWidget->ResetExperiment();
00823       m_pcUserFunctions->Reset();
00824       /* Change state and emit signal */
00825       m_eExperimentState = EXPERIMENT_INITIALIZED;
00826       emit ExperimentReset();
00827    }
00828 
00829    /****************************************/
00830    /****************************************/
00831 
00832    void CQTOpenGLMainWindow::SuspendExperiment() {
00833       /* Toggle action states */
00834       m_pcPlayAction->setEnabled(false);
00835       m_pcResetAction->setEnabled(true);
00836       m_pcTerminateAction->setEnabled(true);
00837       m_pcCaptureAction->setEnabled(false);
00838       m_pcCaptureAction->setChecked(false);
00839       if(! CSimulator::GetInstance().IsRealTimeClock()) {
00840          m_pcPauseAction->setEnabled(false);
00841          m_pcStepAction->setEnabled(false);
00842          m_pcFastForwardAction->setEnabled(false);
00843       }
00844       /* Call OpenGL widget */
00845       m_pcOpenGLWidget->PauseExperiment();
00846       /* Change state and emit signal */
00847       m_eExperimentState = EXPERIMENT_SUSPENDED;
00848       emit ExperimentSuspended();
00849    }
00850 
00851    /****************************************/
00852    /****************************************/
00853 
00854    void CQTOpenGLMainWindow::ResumeExperiment() {
00855       /* Make sure we are in the right state */
00856       if(m_eExperimentState != EXPERIMENT_SUSPENDED) {
00857          LOGERR << "[BUG] CQTOpenGLMainWindow::ResumeExperiment() called in wrong state: "
00858                 << m_eExperimentState
00859                 << std::endl;
00860          LOGERR.Flush();
00861          return;
00862       }
00863       /* Toggle action states */
00864       m_pcPlayAction->setEnabled(true);
00865       m_pcResetAction->setEnabled(false);
00866       m_pcTerminateAction->setEnabled(false);
00867       m_pcCaptureAction->setEnabled(true);
00868       if(! CSimulator::GetInstance().IsRealTimeClock()) {
00869          m_pcStepAction->setEnabled(true);
00870          m_pcFastForwardAction->setEnabled(true);
00871       }
00872       /* Change state and emit signal */
00873       m_eExperimentState = EXPERIMENT_PAUSED;
00874       emit ExperimentResumed();
00875    }
00876 
00877    /****************************************/
00878    /****************************************/
00879 
00880    void CQTOpenGLMainWindow::CameraXMLPopUp() {
00881       /* Set the text window up */
00882       QTextEdit* pcXMLOutput = new QTextEdit();
00883       /* Calculate the geometry of the window so that it's 1/4 of the main window
00884          and placed in the exact center of it */
00885       QRect cGeom = geometry();
00886       cGeom.setBottomRight(geometry().center());
00887       cGeom.moveCenter(geometry().center());
00888       pcXMLOutput->setGeometry(cGeom);
00889       /* This window steals all input */
00890       pcXMLOutput->setWindowModality(Qt::ApplicationModal);
00891       /* You can't modify its contents (but can copy-paste them) */
00892       pcXMLOutput->setReadOnly(true);
00893       /* Set nice document name and window title */
00894       pcXMLOutput->setDocumentTitle("ARGoS XML camera config");
00895       pcXMLOutput->setWindowTitle("ARGoS XML camera config");
00896       /* Set the actual text to visualize */
00897       pcXMLOutput->setPlainText(GetCameraXMLData());
00898       /* Finally, show the resulting window */
00899       pcXMLOutput->show();
00900    }
00901 
00902    /****************************************/
00903    /****************************************/
00904 
00905    // void CQTOpenGLMainWindow::POVRaySceneXMLPopUp() {
00906    //    /* Set the text window up */
00907    //    QTextEdit* pcPOVRayOutput = new QTextEdit();
00908    //    /* Calculate the geometry of the window so that it's 1/4 of the main window
00909    //       and placed in the exact center of it */
00910    //    QRect cGeom = geometry();
00911    //    cGeom.setBottomRight(geometry().center());
00912    //    cGeom.moveCenter(geometry().center());
00913    //    pcPOVRayOutput->setGeometry(cGeom);
00914    //    /* This window steals all input */
00915    //    pcPOVRayOutput->setWindowModality(Qt::ApplicationModal);
00916    //    /* You can't modify its contents (but can copy-paste them) */
00917    //    pcPOVRayOutput->setReadOnly(true);
00918    //    /* Set nice document name and window title */
00919    //    pcPOVRayOutput->setDocumentTitle("ARGoS-POVRay XML camera config");
00920    //    pcPOVRayOutput->setWindowTitle("ARGoS-POVRay XML camera config");
00921    //    /* Set the actual text to visualize */
00922    //    pcPOVRayOutput->setPlainText(GetPOVRaySceneXMLData());
00923    //    /* Finally, show the resulting window */
00924    //    pcPOVRayOutput->show();
00925    // }
00926 
00927    /****************************************/
00928    /****************************************/
00929 
00930    QString CQTOpenGLMainWindow::GetCameraXMLData() {
00931       QString strResult("<camera>\n");
00932       /* Get a reference to the camera */
00933       CQTOpenGLCamera& cCamera = m_pcOpenGLWidget->GetCamera();
00934       for(UInt32 i = 0; i < 12; ++i) {
00935          /* Get its position and target */
00936          const CQTOpenGLCamera::SSettings& sSettings = cCamera.GetSetting(i);
00937          const CVector3& cPos = sSettings.Position;
00938          const CVector3& cLookAt = sSettings.Target;
00939          const CVector3& cUp = sSettings.Up;
00940          strResult.append(
00941             QString("   <placement idx=\"%1\" position=\"%2,%3,%4\" look_at=\"%5,%6,%7\" up=\"%8,%9,%10\" lens_focal_length=\"%11\" />\n")
00942             .arg(i)
00943             .arg(cPos.GetX())
00944             .arg(cPos.GetY())
00945             .arg(cPos.GetZ())
00946             .arg(cLookAt.GetX())
00947             .arg(cLookAt.GetY())
00948             .arg(cLookAt.GetZ())
00949             .arg(cUp.GetX())
00950             .arg(cUp.GetY())
00951             .arg(cUp.GetZ())
00952             .arg(sSettings.LensFocalLength * 1000.0f));
00953       }
00954       strResult.append("</camera>\n");
00955       return strResult;
00956    }
00957 
00958    /****************************************/
00959    /****************************************/
00960 
00961    // QString CQTOpenGLMainWindow::GetPOVRaySceneXMLData() {
00962    //    /* Get the current experiment step */
00963    //    UInt32 unStep = CSimulator::GetInstance().GetSpace().GetExperimentClock();
00964    //    /* Get a reference to the camera */
00965    //    const CQTOpenGLCamera& cCamera = m_pcOpenGLWidget->GetCamera();
00966    //    /* Get its current position and target */
00967    //    const CVector3& cPos = cCamera.GetPosition();
00968    //    const CVector3& cLookAt = cCamera.GetTarget();
00969    //    /* Get the environment node and its contents from the 'povray_render', if defined */
00970    //    TConfigurationNode& tExperiment = CSimulator::GetInstance().GetConfigurationRoot();
00971    //    TConfigurationNode& tVisualization = GetNode(tExperiment, "visualization");
00972    //    QString strPOVRayEnvironment;
00973    //    if(NodeExists(tVisualization,"povray_render")) {
00974    //       TConfigurationNode& tPOVRayVisualization = GetNode(tVisualization, "povray_render");
00975    //       TConfigurationNode& tPOVRayEnvironment = GetNode(tPOVRayVisualization, "environment");
00976    //       std::string strPOVRayEnvironmentNodeContent = tPOVRayEnvironment.ToString(tPOVRayEnvironment);
00977    //       strPOVRayEnvironment = strPOVRayEnvironmentNodeContent.c_str();
00978    //    }
00979 
00980    //    /* Return the XML portion */
00981    //    return QString(
00982    //       "%1\n"
00983    //       "<scene step=\"%2\">\n"
00984    //       "   <camera type=\"normal\"\n"
00985    //       "           position=\"%3,%4,%5\"\n"
00986    //       "           look_at=\"%6,%7,%8\"\n"
00987    //       "           focal_length=\"%9\" />\n"
00988    //       "</scene>\n"
00989    //       )
00990    //       .arg(strPOVRayEnvironment)
00991    //       .arg(unStep)
00992    //       .arg(cPos.GetX())
00993    //       .arg(cPos.GetY())
00994    //       .arg(cPos.GetZ())
00995    //       .arg(cLookAt.GetX())
00996    //       .arg(cLookAt.GetY())
00997    //       .arg(cLookAt.GetZ())
00998    //       .arg(cCamera.GetLensFocalLength() * 1000.0f);
00999    // }
01000 
01001    /****************************************/
01002    /****************************************/
01003 
01004    // void CQTOpenGLMainWindow::POVRayScenePreview() {
01005    //    try {
01006    //       /* Initialize the POV-Ray working directory */
01007    //       QDir cDirectory(QDir::tempPath() + "/argos-povray");
01008    //       /* Erase it if it exists */
01009    //       if(cDirectory.exists()) {
01010    //          if(::system(QString("rm -rf %1").arg(cDirectory.absolutePath()).toAscii().data()) != 0) {
01011    //             THROW_ARGOSEXCEPTION("Could not remove directory \"" <<
01012    //                                  cDirectory.absolutePath().toAscii().data() << "\".");
01013    //          }
01014    //       }
01015    //       /* Create the directory */
01016    //       if(::system(QString("mkdir %1").arg(cDirectory.absolutePath()).toAscii().data()) != 0) {
01017    //          THROW_ARGOSEXCEPTION("Could not create directory \"" <<
01018    //                               cDirectory.absolutePath().toAscii().data() << "\".");
01019    //       }
01020    //       /* Now create the XML file that will contain the POV-Ray scene configuration */
01021    //       QFile cPOVRayXMLConf(cDirectory.absolutePath() + "/argos-povray.xml");
01022    //       cPOVRayXMLConf.open(QFile::WriteOnly | QFile::Truncate);
01023    //       /* Associate a text stream to perform writing to it */
01024    //       QTextStream cPOVRayXMLConfStream(&cPOVRayXMLConf);
01025    //       /* Write the configuration */
01026    //       cPOVRayXMLConfStream << "<povray_render id=\"pov\" output_folder=\"" << cDirectory.absolutePath() << "\">\n";
01027    //       cPOVRayXMLConfStream << GetPOVRaySceneXMLData();
01028    //       cPOVRayXMLConfStream << "</povray_render>\n";
01029    //       cPOVRayXMLConf.close();
01030    //       /* Now parse this file as an ARGoS TConfigurationTree */
01031    //       ticpp::Document tPOVRayXMLConfTree(cPOVRayXMLConf.fileName().toAscii().data());
01032    //       tPOVRayXMLConfTree.LoadFile();
01033    //       /* It's time to create the POV-Ray visualization */
01034    //       CPovrayRender cPOVRayRender;
01035    //       cPOVRayRender.Init(*tPOVRayXMLConfTree.FirstChildElement());
01036    //       /* Write the .pov frame file */
01037    //       cPOVRayRender.WriteOneFrame(cDirectory.absolutePath().append("/pov/frame.pov").toAscii().data());
01038    //       /* Eventually, call POV-Ray to render the file */
01039    //       if(::system(QString("cd %1 && ")
01040    //                   .arg(cDirectory.absolutePath())
01041    //                   .append("./render_single_frame_on_pc.sh pov/frame.pov")
01042    //                   .toAscii().data()) !=0) {
01043    //          THROW_ARGOSEXCEPTION("Could not create POV-Ray preview");
01044    //       }
01045    //    }
01046    //    catch(CARGoSException& ex) {
01047    //       QString strError = QString("Error creating POV-Ray preview\n%1").arg(QString(ex.what()));
01048    //       QMessageBox::critical(this,
01049    //                             tr("ARGoS v2.0"),
01050    //                             strError,
01051    //                             QMessageBox::Ok);
01052    //    }
01053    // }
01054 
01055    /****************************************/
01056    /****************************************/
01057 
01058    void CQTOpenGLMainWindow::SwitchCamera(QAction* pc_action) {
01059       emit CameraSwitched(pc_action->data().toInt());
01060       m_pcFocalLength->setValue(m_pcOpenGLWidget->GetCamera().GetActiveSettings().LensFocalLength * 1000.0f);
01061    }
01062 
01063    /****************************************/
01064    /****************************************/
01065 
01066 }
```

---

Generated on 10 Jul 2018 for ARGoS by 
 1.6.1
